# 

**Published:** 1858-09

**Authors:** 


### CONTENTS.

### *ORIGINAL COMMUNICATIONS.*

PAGE.
ARTICLE I.—On Arsenic in Cutaneous Diseases. By JOHN R. CUSHING, M. D., South Butler, Ala ........ 1
ARTICLE II.—A case of Suppuration of the Bra'n. By WM. H. PHILLPOT, M. D., of Talbot County, Ga,........ 3
ARTICLE III.—Ointment of Iodine and Belladonna in Inflamed Mammæ. By J. S. WEATHERLY, M. D., Montgomery, Ala........ 6
ARTICLE IV.—Hemorrhage from the Extraction of Teeth. By F. D. THURMAN. M. D , Dentist, Atlanta, Geo........ 7
ARTICLE V.—Poetry of Medicine. By J S. SLAUGHTER, Author of Madeline &c., Atlanta, Geo,........ 8
ARTICLE VI.—Infamous attack of the Nashville Journal upon Dr. S. D. Gross, and the Jefferson Medical Col!ege. By MOXA........ 10

### *SELECTIONS.*

Meeting of the Maine Medical Association,........ 18
Practical Remarks on Epigenesis and Sterility,........ 37
Fibrous Tumor of the Uterus complicating Labor,........ 44
American Medical Association,........

### *EDITORIAL AND MISCELLANEOUS.*

Atlanta Medical College—close of the Session,........ 50
Veratrum Viride,........ 54
Bibliographical,........ 55
College Announcements Received,........ 63
"Infamous Attack of the Nashville Journal, upon Dr. Gross and the Jefferson Medical College."........ 64
The Medical Journal of North Carolina,........ 64
Maine Medical and Surgical Reporter,........ 64
Advertisements,........ 64

